# Supplementary material for: ClusterRadar: An interactive web-tool for the multi-method exploration of spatial clusters over time
Source: PLoS One. 2025 May 27;20(5):e0322393. doi: 10.1371/journal.pone.0322393 (PMC12112157; doi:10.1371/journal.pone.0322393)
Supplement: S2 Appendix — Greater detail on how permutation tests are used within ClusterRadar to assess significance. (PDF) [file pone.0322393.s002.pdf]

# S1 Appendix

## Assessing Significance

Permutation testing for spatial statistics involves shuffling data values across locations, calculating the statistic for each shuffle, and constructing an empirical distribution to test the significance of the observed value. This approach differs slightly for global and local spatial statistics: for global statistics, all values are shuffled, for local statistics, the focal location's value is fixed and all other values are shuffled. A pseudo p-value is calculated by first counting the number of permuted values of the statistic which are more extreme than than the actual value:

$$R = \min(|\{s \in S \mid s > v\}|, M - min(|\{s \in S \mid s > v\}|) \quad (1)$$

Where $M$ is the number of permutations performed, $S$ is the set of permuted values for the statistic, and $v$ is the actual value of the statistic on the dataset. The pseudo p-value can now be calculated from $R$ and $M$:

$$p* = \frac{R+1}{M+1} \quad (2)$$

To get the upper and lower significance boundaries, we can re-arrange the $p*$ equation and set $p*$ to a specific p-value cut-off threshold $p* = p_{cutoff}$, giving:

$$R_{cutoff} = \lfloor p_{cutoff} \cdot (M+1) - 1 \rfloor \quad (3)$$

To apply this index, we need $S'$, a numerically sorted list of the permuted values $S$. With $S'$ and $R_{cutoff}$, the calculation for the upper and lower cut-off values for the statistic are:

$$s_{lower} = S'_{R_{cutoff}} \quad (4)$$

$$s_{upper} = S'_{M-R_{cutoff}} \quad (5)$$
