# Supplementary material for: ClusterRadar: An interactive web-tool for the multi-method exploration of spatial clusters over time
Source: PLoS One. 2025 May 27;20(5):e0322393. doi: 10.1371/journal.pone.0322393 (PMC12112157; doi:10.1371/journal.pone.0322393)
Supplement: S3 Appendix — Greater detail on how colors are assigned in ClusterRadar. (PDF) [file pone.0322393.s003.pdf]

S2 Appendix

ClusterRadar Coloring

This appendix provides a detailed description of the coloring schemes used in the visualization tool. It elaborates on the single-indicator color assignments and presents the full mathematical formulation for the aggregate color scheme, including the handling of special cases.

The aggregate color scheme attempts to show, at a glance, the extent to which the different clustering methods agree on their assignments at a location. The scheme employs linear color scales to represent agreement, with bolder, more saturated colors representing a greater degree of agreement and muted, less saturated colors representing a lesser degree of agreement. A location at a specific timestep is first assigned a core group color: red, if a high cluster assignment is present among the assignment set, or blue, if a low cluster assignment is present among the assignment set. If both are present, then the location is a assigned a deep purple color representing a major conflict.

With the core color assigned, the final color is decided by the proportion and nature of non-conflicting contradictory assignments in the assignment set. A non-conflicting contradictory assignment is one which does not strictly fall into the same assignment group as the core assignment, but also does not necessarily contradict it. For example, if a location is assigned "high-high" by Local Moran's I and "not significant" from both Getis-Ord Gi* and Geary's C, then the core assignment group is "high cluster" and each of the "non significant" assignments are treated as non-conflicting contradictory assignments. At timestep t, the assigned color at location i is calculated as follows:

$$\text{color}_{t,i} = \text{col}(g_{t,i}) + h_{t,i} \cdot [\text{col}(g_{t,i}) - \text{col}(\text{"not significant"})] \tag{1}$$

Where $\text{col}(g_{t,i})$ returns the an RGB color space vector representing the color assignment for the core group $g_{t,i}$. The core color assignments are a red color for the "high cluster" group, a blue color for the "low cluster" group, and a light grey for the "not significant" group. The factor $h_{t,i}$ is a scalar encapsulating the degree of non-conflicting disagreement:

$$h_{t,i} = \frac{1}{|L|} \cdot \sum_{l \in \mathrm{L}_{t,i}} d(g_{t,i}, l) \tag{2}$$

Where $\mathrm{L}_{t,i}$ is the set of cluster assignments for location i at time step t, and $d(g, l)$ returns a number between 0 and 1 representing the extent of disagreement

between core group g and assignment l. The extent of disagreement is $d(g,l) = 0$ for labels which belong in the group and $d(g,l) = 1$ for non-conflicting contradictory labels of the group. There is also a special case of $d(g,l) = 0.5$ when the core group is "high cluster" or "low cluster" and the the assignment l is "other positive spatial autocorrelation". This assignment is from the Geary's C method when it recognizes positive spatial autocorrelation but is unable to determine its exact nature — a quirk of the method. It does not necessarily contradict either of the positive core group assignments, but it is not a confident agreement either, hence our decision to assign it "partial" agreement.

There are three special cases. The first is the aforementioned major conflict case where two of the assigned labels at a location belong to conflicting groups — this is assigned a deep purple. Sometimes assignments occur which, depending on how the methods are interpreted, can be considered a less serious conflict. For example, if Local Moran's I assigns "low-high" and Getis-Ord Gi* assigns "hot-spot", then both assignments recognize a potential positive clustering of high valued neighbors, but there is a potential disagreement on the overall assignment. The third special case is when only miscellaneous assignments and "not-significant" assignments are found in the assigned labels. For a graphical explanation of the aggregate color scheme, see Fig. 1.

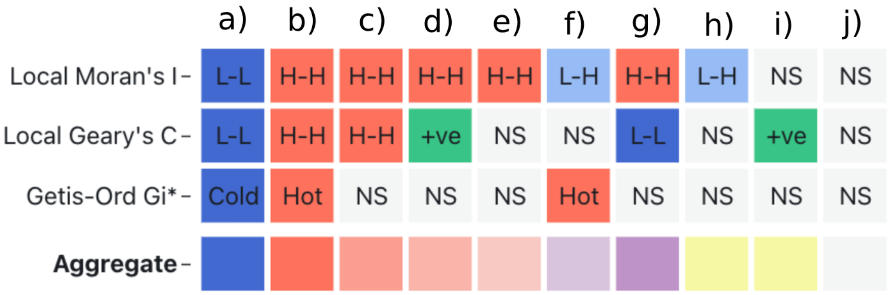

Figure 1: Examples of how colors are assigned using the individual and aggregate color schemes. This plot shows examples of how the individual cluster assignments are aggregated into a single aggregate color. The aggregate color scheme visually represents the level of agreement between clustering methods. Locations are assigned a core color: red for "high-high" or "hot-spot", blue for "low-low" or "cold-spot". The final color is adjusted based on the presence of non-conflicting assignments: assignments that don't match the core assignment but don't directly contradict it either. A purple color is used for conflicts: a deeper purple for more significant conflicts. A yellow color is used for other assignments that do not fall naturally into the high or low cluster groups. Examples in this plot include total agreement in the main positive groups (a and b), partial agreement (c, d, e), minor conflict (f), major conflict (g), other (h,i) and not-significant (j).
