# Supplementary material for: ClusterRadar: An interactive web-tool for the multi-method exploration of spatial clusters over time
Source: PLoS One. 2025 May 27;20(5):e0322393. doi: 10.1371/journal.pone.0322393 (PMC12112157; doi:10.1371/journal.pone.0322393)
Supplement: S4 Appendix — Performance benchmarks for ClusterRadar. (PDF) [file pone.0322393.s004.pdf]

S3 Appendix

ClusterRadar Performance

Currently, jsgeoda[1] (a WebAssembly compilation of the libgeoda C++ library) is the only web-based library we found that supports the spatial autocorrelation methods required for ClusterRadar. This appendix compares the performance of ClusterRadar's local spatial autocorrelation calculations (its most computationally intensive task) to the corresponding calculations in jsgeoda to ensure the performance of our JavaScript implementation remains practical for intended use cases. All performance comparisons used artificially generated GeoJSON grid data, with permutation testing performed using the standard default of 999 permutations. ClusterRadar requires additional calculations beyond those provided by jsgeoda, notably the calculation of significance cutoff values and the estimation of statistic distributions (using kernel density estimation). To directly compare the core operations, we first disable these features in ClusterRadar (see Fig. 1). We then compare this simplified pipeline to the full pipeline (Fig. 2). Finally, we show the performance of the complete, default three-method pipeline across a wider range of simulated GeoJSON sizes (Fig. 3).

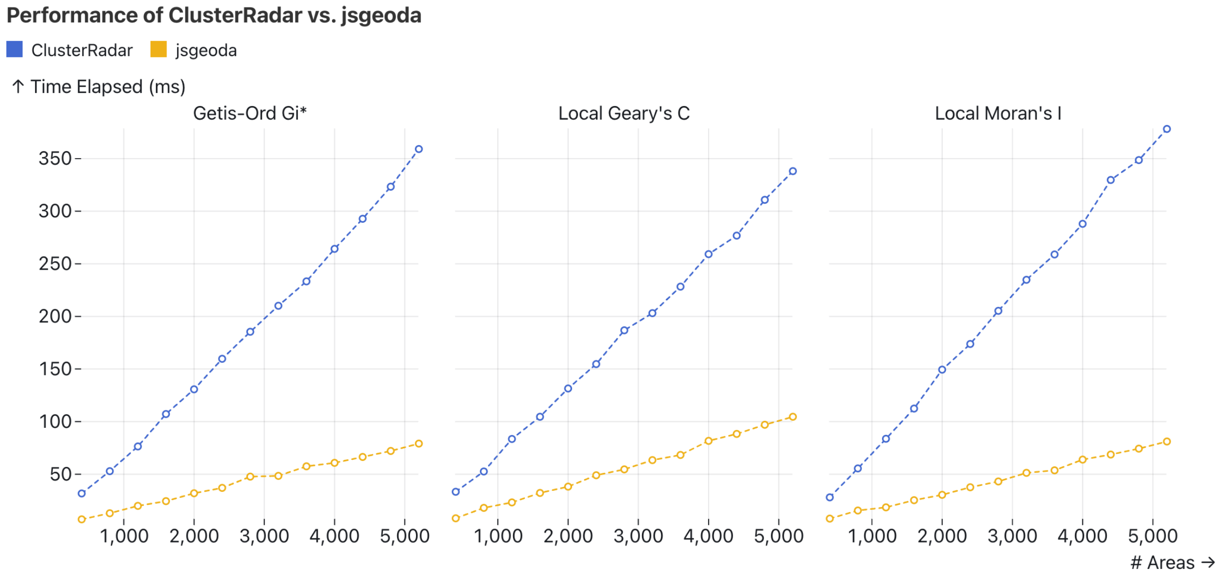

Figure 1: Comparison of execution time for ClusterRadar and jsgeoda calculating the three Local Indicators of Spatial Association (LISAs) enabled by default in ClusterRadar. The ClusterRadar pipeline was simplified to achieve closer functional equivalence with jsgeoda.

[1] jsgeoda's source code can be found at `https://github.com/GeoDaCenter/jsgeoda`

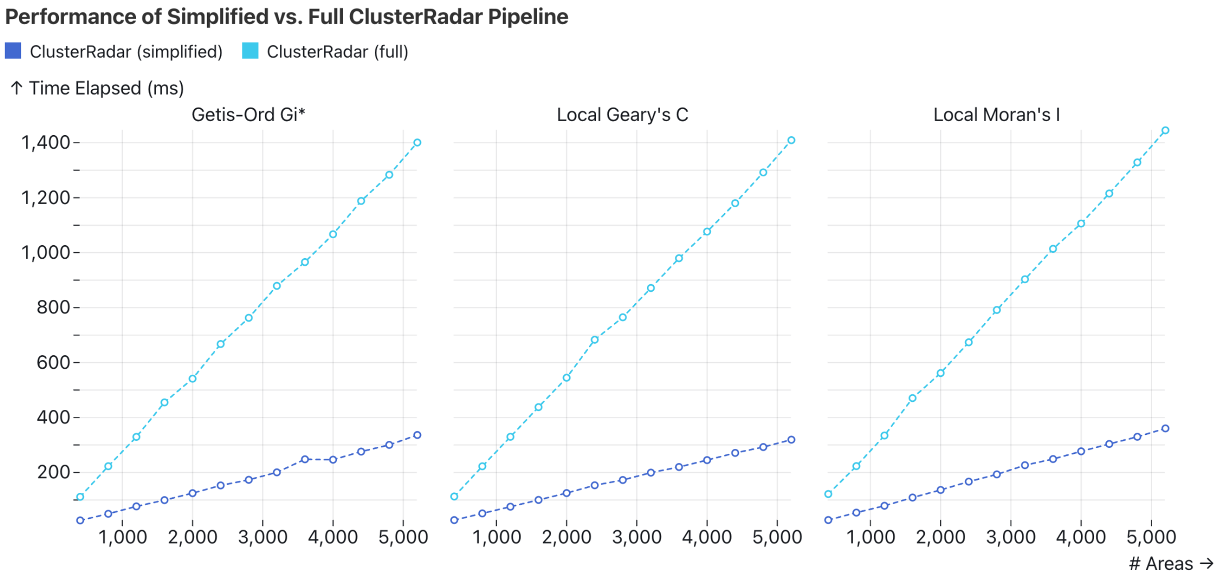

Figure 2: Performance comparison between a minimal LISA calculation and the full analytical pipeline used by ClusterRadar. The minimal calculation, similar in functionality to other LISA libraries, computes LISAs and their pseudo p-values via permutation tests. The full ClusterRadar pipeline adds two crucial steps for each location: calculation of high and low critical values (significance thresholds), and estimation of the permuted statistic distribution using kernel density estimation (KDE). These additional steps are not standard in typical LISA calculation libraries but are necessary for key visualizations within ClusterRadar.

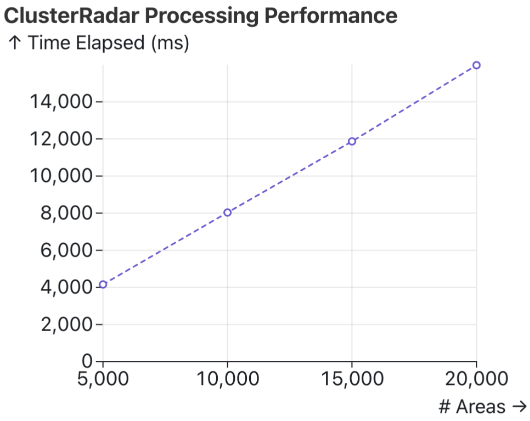

Figure 3: Performance of ClusterRadar's full analytical pipeline, encompassing all three LISA methods enabled by default in the ClusterRadar web-tool.
