# Supplementary material for: ClusterRadar: An interactive web-tool for the multi-method exploration of spatial clusters over time
Source: PLoS One. 2025 May 27;20(5):e0322393. doi: 10.1371/journal.pone.0322393 (PMC12112157; doi:10.1371/journal.pone.0322393)
Supplement: S5 Appendix — Memory usage benchmarks for ClusterRadar. (PDF) [file pone.0322393.s005.pdf]

S4 Appendix

ClusterRadar Memory Usage

Available memory in the browser is typically more limited than in a native application, and therefore browser applications need to be cautious about memory use. Exact limits are variable based on a number of factors such as the browser being used and the user's specific settings. We have performed a quick evaluation of ClusterRadar's analytical pipeline memory usage. The results are shown in Fig. 1.

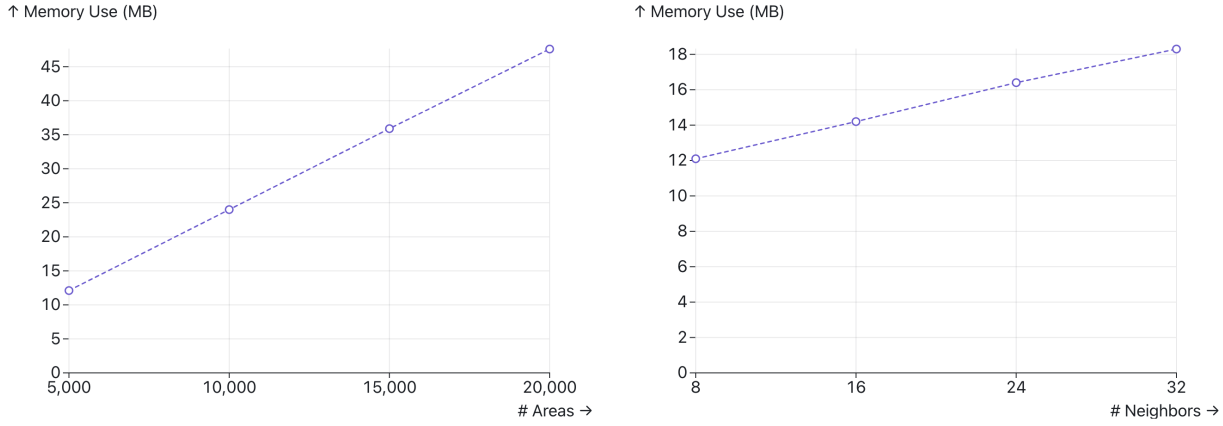

Figure 1: Memory use of ClusterRadar on the default processing pipeline comprised of three methods. Memory use is evaluated by number of areas (left) and by number of neighbors (right). In the former evaluation, the number of neighbors is fixed at 8, and in the latter evaluation, the number of areas is fixed at 5,000. These results are for a single timestep. If multiple timesteps are present, these numbers will scale linearly with the number of timesteps, limited by the number of threads used at once. If four threads are used, the total memory usage of ClusterRadar for a 20,000 area dataset with a mean of 8 neighbors per area will be approximately 190.4 MB. The graph shows that memory use scales linearly with the number of areas and the number of neighbors in the dataset.

The memory usage profiled in Fig. 1 is for the isolated analytical pipeline, and not the entire dashboard tool. Memory usage of the dashboard depends on some additional factors, including the resolution of the GeoJSON data file and (less significantly) on the complexity of plots, determined primarily by the number of timesteps in the data. We profiled the full ClusterRadar dashboard on the full default cancer mortality dataset, comprised of 3,143 areas and 22 time-points. The application used 144 MB of memory in total. This evaluation shows that ClusterRadar does not use an excessive amount of memory, and memory use scales linearly, but for very large datasets (e.g. US census block level data, comprised of over 8million areas) ClusterRadar may become infeasible in current browsers.
