# Supplementary material for: ClusterRadar: An interactive web-tool for the multi-method exploration of spatial clusters over time
Source: PLoS One. 2025 May 27;20(5):e0322393. doi: 10.1371/journal.pone.0322393 (PMC12112157; doi:10.1371/journal.pone.0322393)
Supplement: S6 File — (PDF) [file pone.0322393.s006.pdf]

ClusterRadar Feedback

This is a feedback form for ClusterRadar that should take about 5-10 minutes. If you'd like to suggest a feature or report a bug, we recommend making an issue on the GitHub repository.

If you'd like to provide some general feedback, feel free to skip to the final page of this form and fill in the "Additional comments and suggestions" box. All feedback and suggestions are really appreciated!

Background

Before using ClusterRadar, what extent were you familiar with the following concepts and techniques?

	Not familiar	Basic understanding	Working knowledge	Expert knowledge
Spatial data and analysis	○	◉	○	○
Analysis of spatial clusters	◉	○	○	○
Local spatial autocorrelation	◉	○	○	○
Hot-spot / cold-spot analysis	◉	○	○	○
Local Moran's I	◉	○	○	○
Local Geary's C	◉	○	○	○
Getis-Ord G/G*	◉	○	○	○

Do you have any additional comments about your familiarity / experience with the concepts listed in the previous question?

At the beginning of the tutorial, or perhaps you could create an “about” page, it would be really useful to have a high-level description of clusters and what we can gain from exploring them. Spatial clusters are regions of space that exhibit similar values. You would expect regions to have similar values if the estimate is based on that region. I.e., if you only have county level data, the county will have similar values. I think that up front you need to define the smallest regions for which there are data. That seems clear from the map label, but I would explicitly say it. Click on the "x" that appears to take the location out of focus. I’m not sure where the x is. I had a hard time interpreting the color values on the time scales at first. A general example would go a long way towards clarifying them. For e.g., the red counties in the northeast indicate high clustering of cancer mortality in 2020 (if that’s correct!!). Zoomed map reel is very cool. But once I selected a few counties I couldn’t figure out how to deselect them and go back to the entire map. Time slider - Density plots – sometimes the red line isn’t within the two dashed lines. For density plot, cell plot, I realize the description box is right above the plots, but could you include “below” just to make it clear? Also, if you move the view from not showing the 3 figures at the bottom back to showing them, the density plot tutorial box is on top of the figure. Could you add information boxes (i with the circle) to each of the figures at the bottom so that you don’t have to go back to the tutorial to determine what they are? For the 3 figures at the bottom, what are the x-axis values for the density and cell plots? What does the correlation box mean? On the right top of the screen, when you hover over the icons (including the github octicat), they don’t show labels.

General Evaluation

To be filled in after using ClusterRadar. General questions about the type of analysis facilitated by ClusterRadar.

How helpful do you feel the analysis of spatial clusters would be for your work?

- ○ Very helpful
- ○ Somewhat helpful
- ◉ Not helpful

Do you feel the analysis of spatial clusters over time provides useful additional insight over the static analysis of spatial clusters? If so, do you feel this is worth the additional complexity?

- (x) Yes, and it is worth the additional complexity
- ( ) Potentially, but it may not be worth the additional complexity
- ( ) No
- ( ) Other:

Do you feel that the simultaneous application and comparison of multiple spatial clustering methods provides useful additional insight over analysis that only employs a single method? If so, do you feel this is worth the additional complexity?

- ( ) Yes, and it is worth the additional complexity
- (x) Potentially, but it may not be worth the additional complexity
- ( ) No
- ( ) Other:

Do you have any additional comments regarding the questions asked in this section?

Just because I probably wouldn't use this because of the type of research I do doesn't mean that I think it wouldn't be useful for others.

ClusterRadar Evaluation

To be filled in after using ClusterRadar. Questions about the specific features and design decisions employed by ClusterRadar.

Do you feel that ClusterRadar achieves its primary goal of making the analysis of spatial clusters over time more accessible?

- ( ) Yes
- (•) Somewhat
- ( ) No

Do you have any additional comments regarding how well ClusterRadar achieved its primary goal?

I answered somewhat above because ClusterRadar seems descriptive at this point, not an analytic tool.

When using ClusterRadar, did you discover any interesting patterns in the data that may warrant further investigation?

How useful did you find the following features of ClusterRadar? A "detrimental" feature is one you found confusing, distracting, or otherwise detrimental to the overall experience of using the tool.

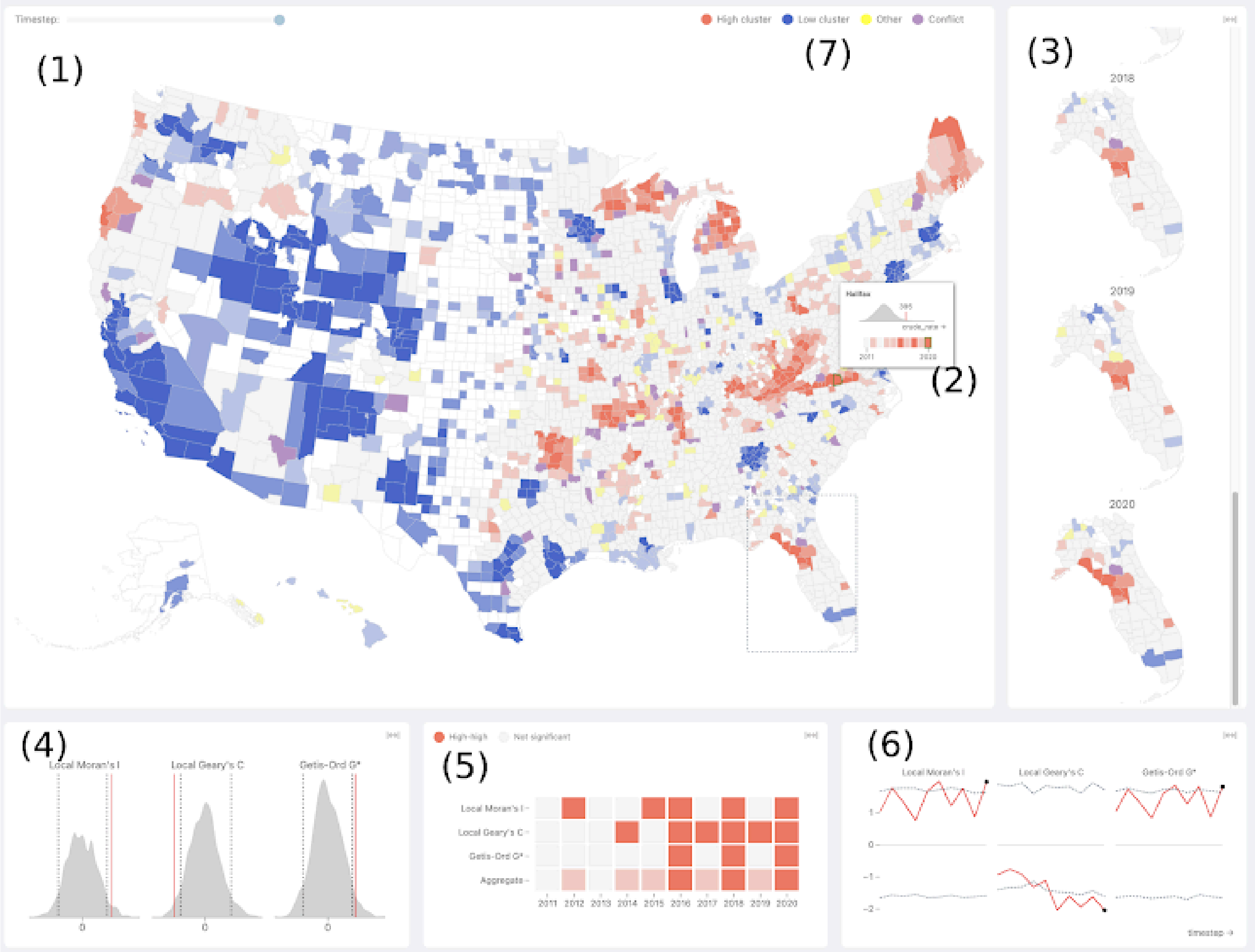

	Very useful	Somewhat useful	Not useful	Detrimental
(1) The main interactive map and time slider (the largest panel).	◉	○	○	○
(2) The graphical tooltip	◉	○	○	○
(3) The zoomed map "reel" showing the evolution of clusters over time (on the right of the page)	◉	○	○	○
(4) The statistical				

(4) The statistical density/distribution plots (bottom left)	○	◉	○	○
(5) The cell plot comparing all methods over time (bottom center)	◉	○	○	○
(6) The time-series plots showing the evolution of the statistic over time with significance cut-off boundaries (bottom right)	◉	○	○	○
(7) The aggregated coloring scheme, showing the extent to which the different methods agreed (default coloring scheme used in the maps)	◉	○	○	○
The tool's design as an in-browser web-tool	◉	○	○	○

Do you have any additional comments or suggestions about ClusterRadar?

I gave 4 a somewhat useful because I didn't really know how to interpret the density plots.

This content is neither created nor endorsed by Google.

Google Forms

ClusterRadar Feedback

This is a feedback form for ClusterRadar that should take about 5-10 minutes. If you'd like to suggest a feature or report a bug, we recommend making an issue on the GitHub repository.

If you'd like to provide some general feedback, feel free to skip to the final page of this form and fill in the "Additional comments and suggestions" box. All feedback and suggestions are really appreciated!

Background

Before using ClusterRadar, what extent were you familiar with the following concepts and techniques?

	Not familiar	Basic understanding	Working knowledge	Expert knowledge
Spatial data and analysis	○	◉	○	○
Analysis of spatial clusters	◉	○	○	○
Local spatial autocorrelation	◉	○	○	○
Hot-spot / cold-spot analysis	○	◉	○	○
Local Moran's I	◉	○	○	○
Local Geary's C	◉	○	○	○
Getis-Ord G/G*	◉	○	○	○

Do you have any additional comments about your familiarity / experience with the concepts listed in the previous question?

No

General Evaluation

To be filled in after using ClusterRadar. General questions about the type of analysis facilitated by ClusterRadar.

How helpful do you feel the analysis of spatial clusters would be for your work?

- ( ) Very helpful
- (•) Somewhat helpful
- ( ) Not helpful

Do you feel the analysis of spatial clusters over time provides useful additional insight over the static analysis of spatial clusters? If so, do you feel this is worth the additional complexity?

- ( ) Yes, and it is worth the additional complexity
- (•) Potentially, but it may not be worth the additional complexity
- ( ) No
- ( ) Other:

Do you feel that the simultaneous application and comparison of multiple spatial clustering methods provides useful additional insight over analysis that only employs a single method? If so, do you feel this is worth the additional complexity?

- [x] Yes, and it is worth the additional complexity
- [ ] Potentially, but it may not be worth the additional complexity
- [ ] No
- [ ] Other: ____________

Do you have any additional comments regarding the questions asked in this section?

____

ClusterRadar Evaluation

To be filled in after using ClusterRadar. Questions about the specific features and design decisions employed by ClusterRadar.

Do you feel that ClusterRadar achieves its primary goal of making the analysis of spatial clusters over time more accessible?

- [ ] Yes
- [x] Somewhat
- [ ] No

Do you have any additional comments regarding how well ClusterRadar achieved its primary goal?

Maybe it is coming, but I would want some basic information about what the interpretation of each method is and how they differ.

When using ClusterRadar, did you discover any interesting patterns in the data that may warrant further investigation?

How useful did you find the following features of ClusterRadar? A "detrimental" feature is one you found confusing, distracting, or otherwise detrimental to the overall experience of using the tool.

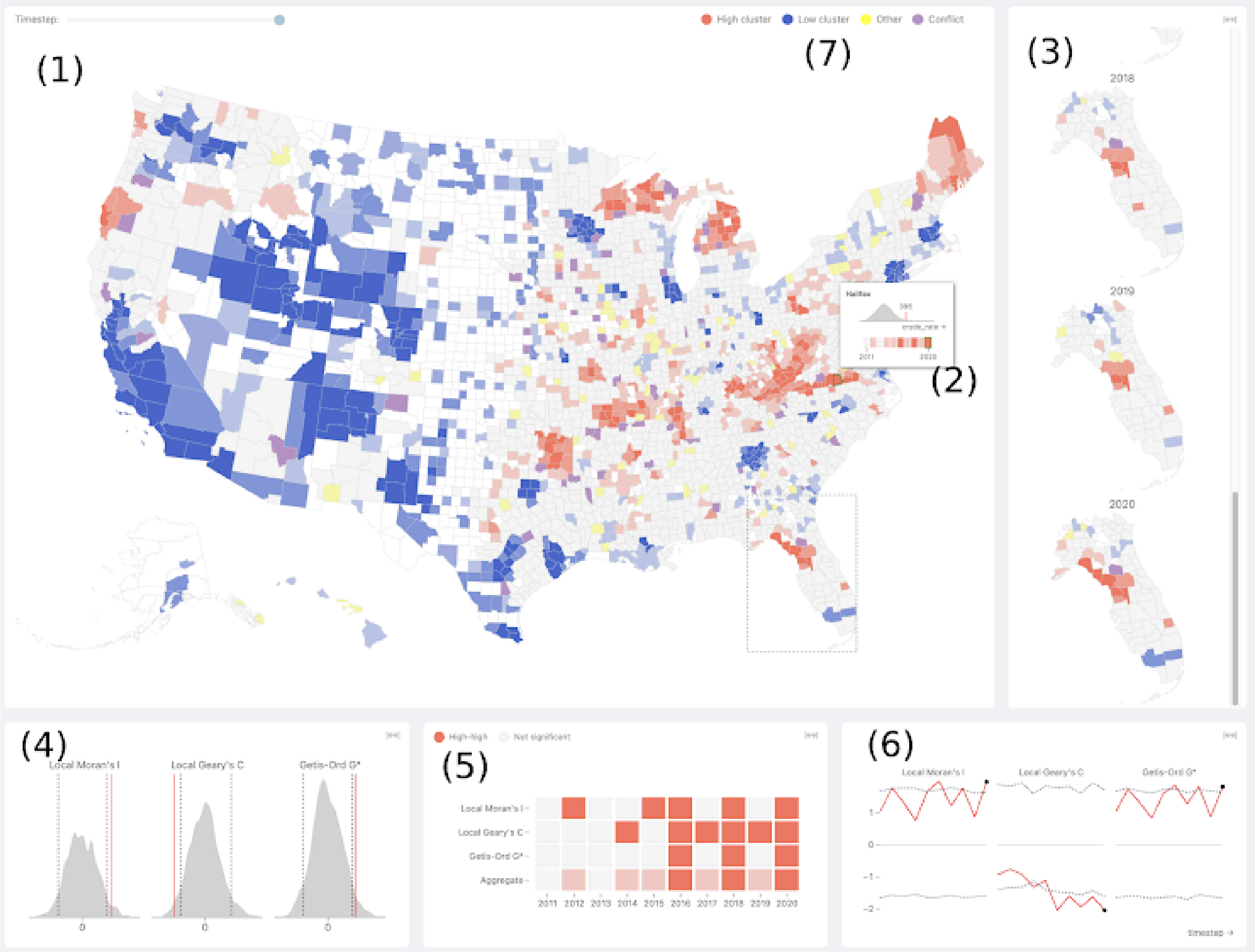

	Very useful	Somewhat useful	Not useful	Detrimental
(1) The main interactive map and time slider (the largest panel).	◉	○	○	○
(2) The graphical tooltip	◉	○	○	○
(3) The zoomed map "reel" showing the evolution of clusters over time (on the right of the page)	○	◉	○	○

(4) The statistical

(4) The statistical density/distribution plots (bottom left)	○	○	◉	○
(5) The cell plot comparing all methods over time (bottom center)	○	◉	○	○
(6) The time-series plots showing the evolution of the statistic over time with significance cut-off boundaries (bottom right)	○	◉	○	○
(7) The aggregated coloring scheme, showing the extent to which the different methods agreed (default coloring scheme used in the maps)	○	◉	○	○
The tool's design as an in-browser web-tool	◉	○	○	○

Do you have any additional comments or suggestions about ClusterRadar?

It was unclear to me how to select the areas that are zoomed in on the right. Are these predetermined and if we click in any area it selects a default section of the graph?

This content is neither created nor endorsed by Google.

Google Forms

ClusterRadar Feedback

This is a feedback form for ClusterRadar that should take about 5-10 minutes. If you'd like to suggest a feature or report a bug, we recommend making an issue on the GitHub repository.

If you'd like to provide some general feedback, feel free to skip to the final page of this form and fill in the "Additional comments and suggestions" box. All feedback and suggestions are really appreciated!

Background

Before using ClusterRadar, what extent were you familiar with the following concepts and techniques?

	Not familiar	Basic understanding	Working knowledge	Expert knowledge
Spatial data and analysis	○	◉	○	○
Analysis of spatial clusters	○	◉	○	○
Local spatial autocorrelation	○	◉	○	○
Hot-spot / cold-spot analysis	◉	○	○	○
Local Moran's I	○	◉	○	○
Local Geary's C	○	◉	○	○
Getis-Ord G/G*	◉	○	○	○

Do you have any additional comments about your familiarity / experience with the concepts listed in the previous question?

General Evaluation

To be filled in after using ClusterRadar. General questions about the type of analysis facilitated by ClusterRadar.

How helpful do you feel the analysis of spatial clusters would be for your work?

- (●) Very helpful
- ( ) Somewhat helpful
- ( ) Not helpful

Do you feel the analysis of spatial clusters over time provides useful additional insight over the static analysis of spatial clusters? If so, do you feel this is worth the additional complexity?

- (●) Yes, and it is worth the additional complexity
- ( ) Potentially, but it may not be worth the additional complexity
- ( ) No
- ( ) Other:

Do you feel that the simultaneous application and comparison of multiple spatial clustering methods provides useful additional insight over analysis that only employs a single method? If so, do you feel this is worth the additional complexity?

- (●) Yes, and it is worth the additional complexity
- ( ) Potentially, but it may not be worth the additional complexity
- ( ) No
- ( ) Other: ____________

Do you have any additional comments regarding the questions asked in this section?

____

ClusterRadar Evaluation

To be filled in after using ClusterRadar. Questions about the specific features and design decisions employed by ClusterRadar.

Do you feel that ClusterRadar achieves its primary goal of making the analysis of spatial clusters over time more accessible?

- (●) Yes
- ( ) Somewhat
- ( ) No

Do you have any additional comments regarding how well ClusterRadar achieved its primary goal?

The panel choices were very insightful. One way to improve would be to provide hover text to explain the measures and what low and high values correspond to. Getis-Ord G* was particularly challenging to understand without having any prior background.

When using ClusterRadar, did you discover any interesting patterns in the data that may warrant further investigation?

How useful did you find the following features of ClusterRadar? A "detrimental" feature is one you found confusing, distracting, or otherwise detrimental to the overall experience of using the tool.

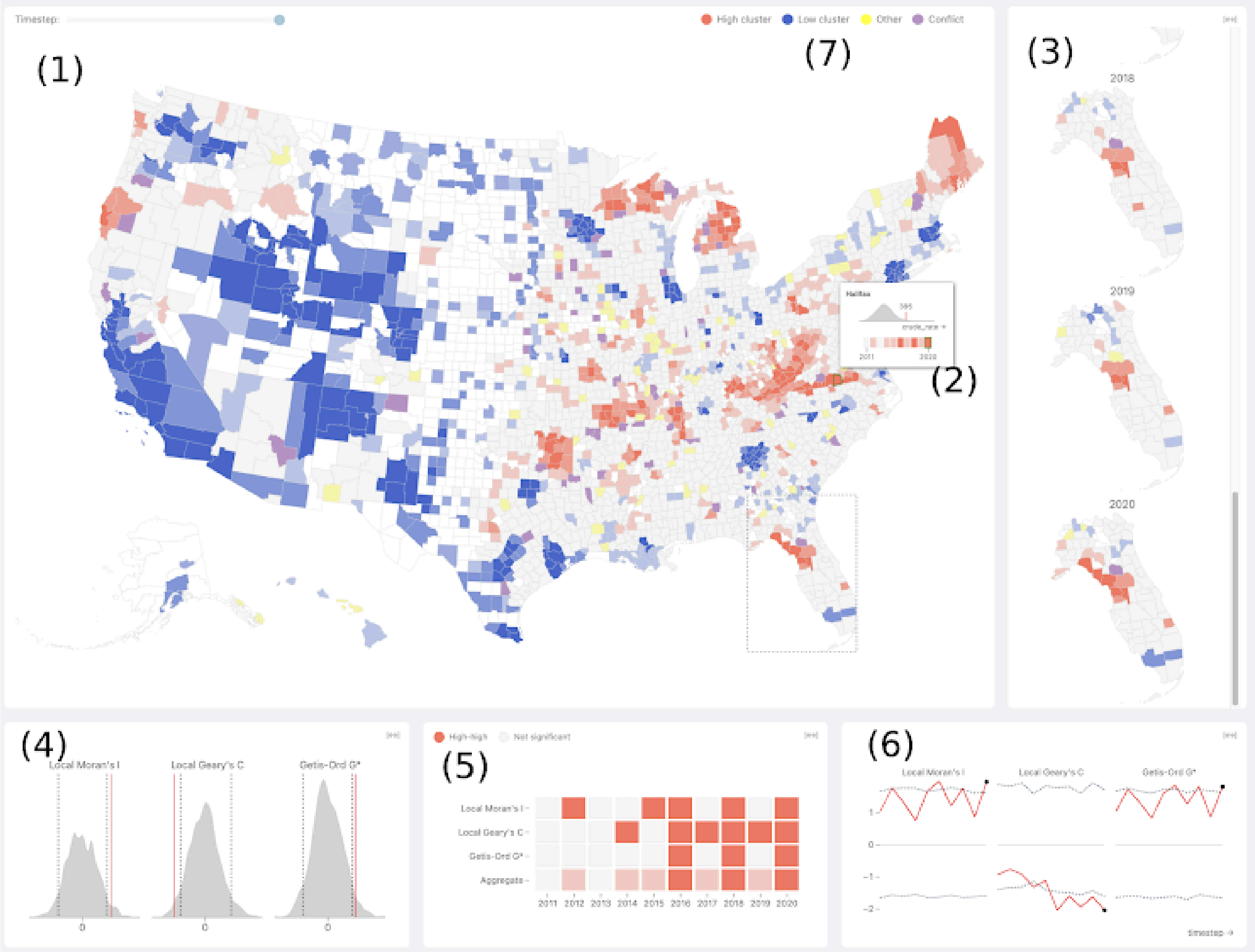

	Very useful	Somewhat useful	Not useful	Detrimental
(1) The main interactive map and time slider (the largest panel).	◉	○	○	○
(2) The graphical tooltip	◉	○	○	○
(3) The zoomed map "reel" showing the evolution of clusters over time (on the right of the page)	◉	○	○	○

(4) The statistical

(4) The statistical density/distribution plots (bottom left)	◉	○	○	○
(5) The cell plot comparing all methods over time (bottom center)	◉	○	○	○
(6) The time-series plots showing the evolution of the statistic over time with significance cut-off boundaries (bottom right)	◉	○	○	○
(7) The aggregated coloring scheme, showing the extent to which the different methods agreed (default coloring scheme used in the maps)	◉	○	○	○
The tool's design as an in-browser web-tool	◉	○	○	○

Do you have any additional comments or suggestions about ClusterRadar?

This content is neither created nor endorsed by Google.

Google Forms

ClusterRadar Feedback

This is a feedback form for ClusterRadar that should take about 5-10 minutes. If you'd like to suggest a feature or report a bug, we recommend making an issue on the GitHub repository.

If you'd like to provide some general feedback, feel free to skip to the final page of this form and fill in the "Additional comments and suggestions" box. All feedback and suggestions are really appreciated!

Background

Before using ClusterRadar, what extent were you familiar with the following concepts and techniques?

	Not familiar	Basic understanding	Working knowledge	Expert knowledge
Spatial data and analysis	○	◉	○	○
Analysis of spatial clusters	○	◉	○	○
Local spatial autocorrelation	○	◉	○	○
Hot-spot / cold-spot analysis	◉	○	○	○
Local Moran's I	◉	○	○	○
Local Geary's C	◉	○	○	○
Getis-Ord G/G*	◉	○	○	○

Do you have any additional comments about your familiarity / experience with the concepts listed in the previous question?

General Evaluation

To be filled in after using ClusterRadar. General questions about the type of analysis facilitated by ClusterRadar.

How helpful do you feel the analysis of spatial clusters would be for your work?

- ( ) Very helpful
- (•) Somewhat helpful
- ( ) Not helpful

Do you feel the analysis of spatial clusters over time provides useful additional insight over the static analysis of spatial clusters? If so, do you feel this is worth the additional complexity?

- (•) Yes, and it is worth the additional complexity
- ( ) Potentially, but it may not be worth the additional complexity
- ( ) No
- ( ) Other:

Do you feel that the simultaneous application and comparison of multiple spatial clustering methods provides useful additional insight over analysis that only employs a single method? If so, do you feel this is worth the additional complexity?

- ○ Yes, and it is worth the additional complexity
- ◉ Potentially, but it may not be worth the additional complexity
- ○ No
- ○ Other: ______

Do you have any additional comments regarding the questions asked in this section?

______

ClusterRadar Evaluation

To be filled in after using ClusterRadar. Questions about the specific features and design decisions employed by ClusterRadar.

Do you feel that ClusterRadar achieves its primary goal of making the analysis of spatial clusters over time more accessible?

- ◉ Yes
- ○ Somewhat
- ○ No

Do you have any additional comments regarding how well ClusterRadar achieved its primary goal?

When using ClusterRadar, did you discover any interesting patterns in the data that may warrant further investigation?

How useful did you find the following features of ClusterRadar? A "detrimental" feature is one you found confusing, distracting, or otherwise detrimental to the overall experience of using the tool.

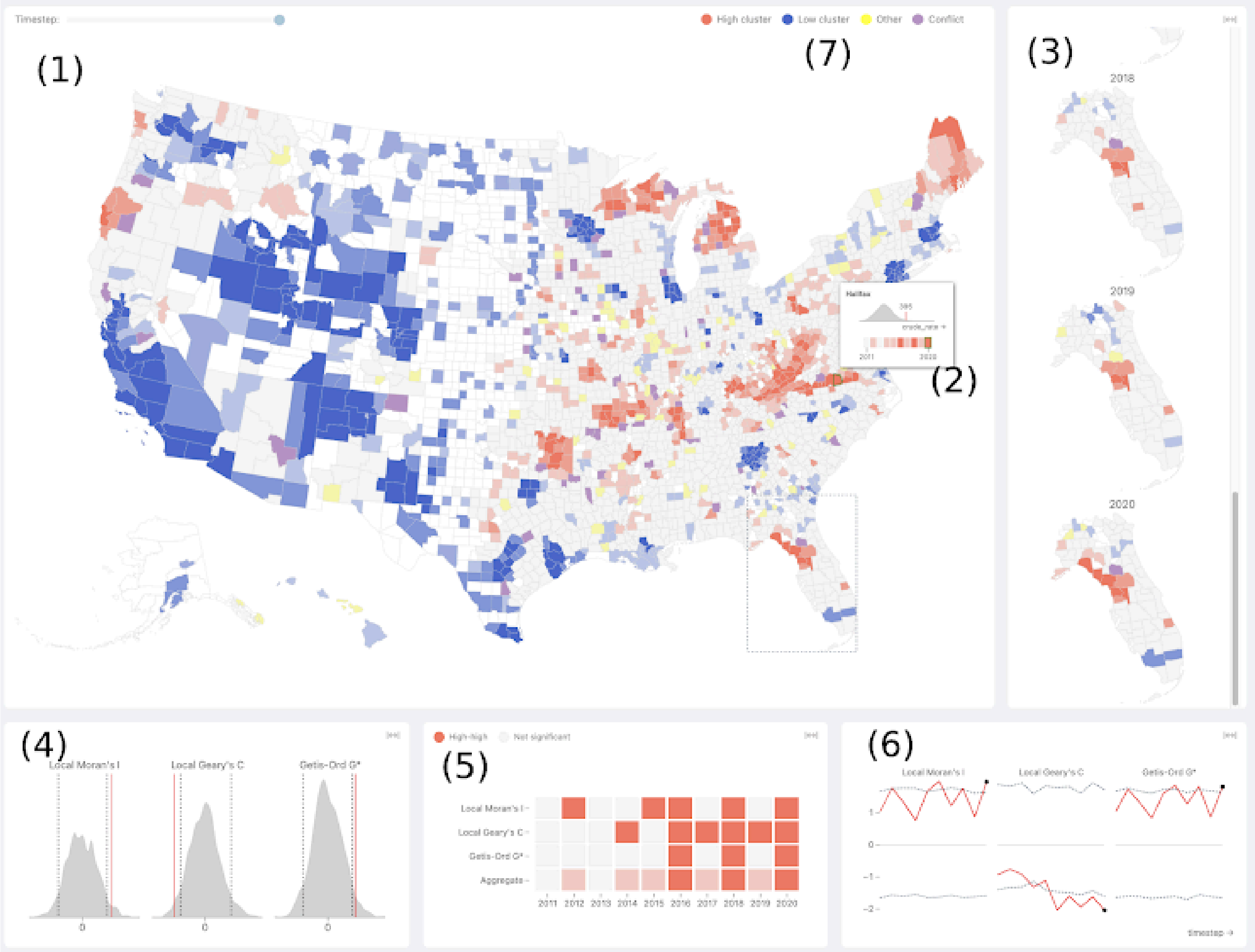

	Very useful	Somewhat useful	Not useful	Detrimental
(1) The main interactive map and time slider (the largest panel).	◉	○	○	○
(2) The graphical tooltip	◉	○	○	○
(3) The zoomed map "reel" showing the evolution of clusters over time (on the right of the page)	◉	○	○	○

(4) The statistical

(4) The statistical density/distribution plots (bottom left)	◉	○	○	○
(5) The cell plot comparing all methods over time (bottom center)	◉	○	○	○
(6) The time-series plots showing the evolution of the statistic over time with significance cut-off boundaries (bottom right)	◉	○	○	○
(7) The aggregated coloring scheme, showing the extent to which the different methods agreed (default coloring scheme used in the maps)	◉	○	○	○
The tool's design as an in-browser web-tool	◉	○	○	○

Do you have any additional comments or suggestions about ClusterRadar?

This content is neither created nor endorsed by Google.

Google Forms

ClusterRadar Feedback

This is a feedback form for ClusterRadar that should take about 5-10 minutes. If you'd like to suggest a feature or report a bug, we recommend making an issue on the GitHub repository.

If you'd like to provide some general feedback, feel free to skip to the final page of this form and fill in the "Additional comments and suggestions" box. All feedback and suggestions are really appreciated!

Background

Before using ClusterRadar, what extent were you familiar with the following concepts and techniques?

	Not familiar	Basic understanding	Working knowledge	Expert knowledge
Spatial data and analysis	○	◉	○	○
Analysis of spatial clusters	○	◉	○	○
Local spatial autocorrelation	○	◉	○	○
Hot-spot / cold-spot analysis	○	◉	○	○
Local Moran's I	○	◉	○	○
Local Geary's C	○	◉	○	○
Getis-Ord G/G*	○	◉	○	○

Do you have any additional comments about your familiarity / experience with the concepts listed in the previous question?

General Evaluation

To be filled in after using ClusterRadar. General questions about the type of analysis facilitated by ClusterRadar.

How helpful do you feel the analysis of spatial clusters would be for your work?

- (●) Very helpful
- ( ) Somewhat helpful
- ( ) Not helpful

Do you feel the analysis of spatial clusters over time provides useful additional insight over the static analysis of spatial clusters? If so, do you feel this is worth the additional complexity?

- (●) Yes, and it is worth the additional complexity
- ( ) Potentially, but it may not be worth the additional complexity
- ( ) No
- ( ) Other:

Do you feel that the simultaneous application and comparison of multiple spatial clustering methods provides useful additional insight over analysis that only employs a single method? If so, do you feel this is worth the additional complexity?

- ○ Yes, and it is worth the additional complexity
- ○ Potentially, but it may not be worth the additional complexity
- ○ No
- ◉ Other: i do not know. i remain uncertain. For playing around with the tool i enjoyed this, and during my studies learning about the G Ortis cluster this would have been great. Sorry for long nonanswer

Do you have any additional comments regarding the questions asked in this section?

ClusterRadar Evaluation

To be filled in after using ClusterRadar. Questions about the specific features and design decisions employed by ClusterRadar.

Do you feel that ClusterRadar achieves its primary goal of making the analysis of spatial clusters over time more accessible?

- ◉ Yes
- ○ Somewhat
- ○ No

Do you have any additional comments regarding how well ClusterRadar achieved its primary goal?

The "Methods" tab link does not work.

When using ClusterRadar, did you discover any interesting patterns in the data that may warrant further investigation?

How useful did you find the following features of ClusterRadar? A "detrimental" feature is one you found confusing, distracting, or otherwise detrimental to the overall experience of using the tool.

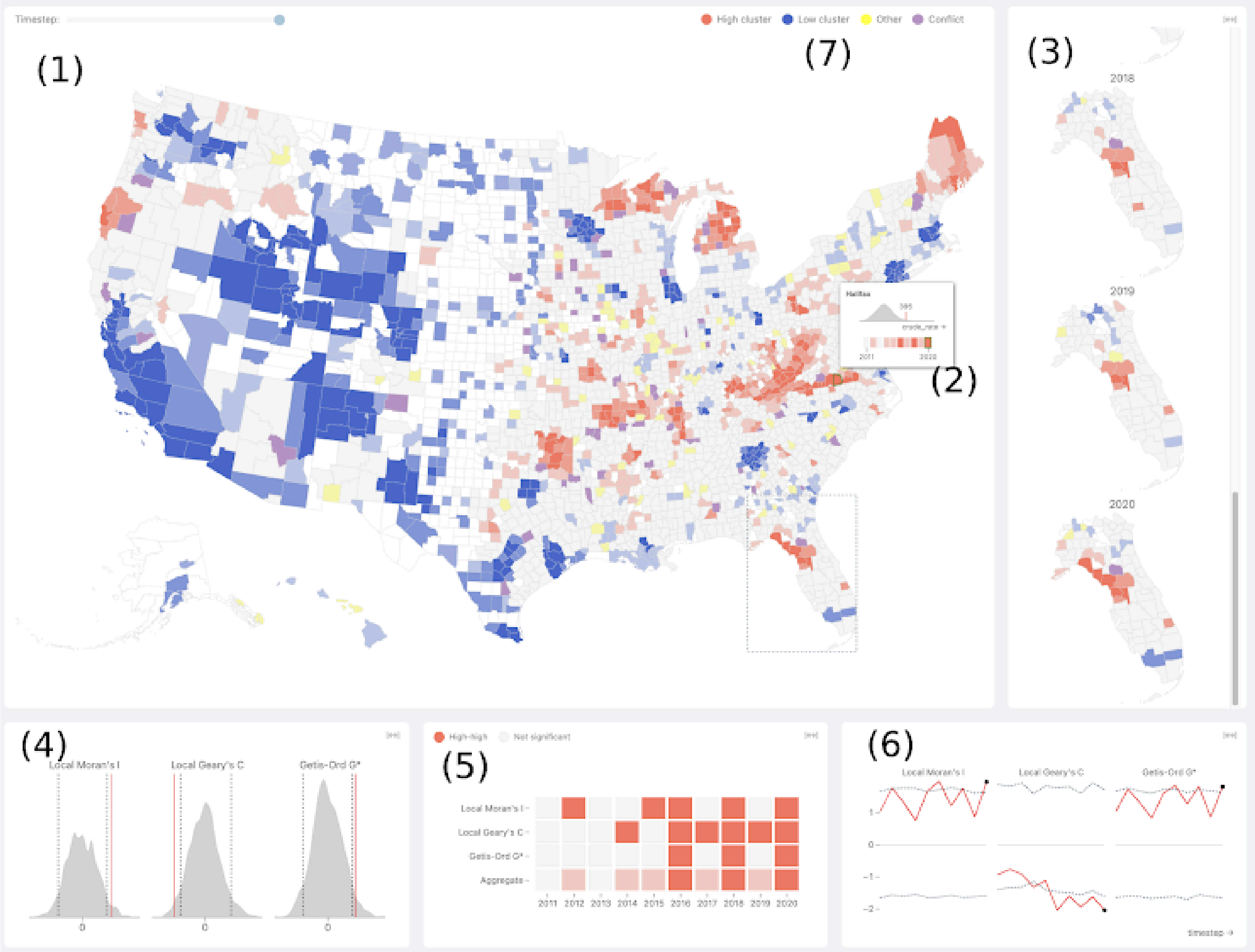

	Very useful	Somewhat useful	Not useful	Detrimental
(1) The main interactive map and time slider (the largest panel).	◉	○	○	○
(2) The graphical tooltip	◉	○	○	○
(3) The zoomed map "reel" showing the evolution of clusters over time (on the right of the page)	◉	○	○	○

(4) The statistical

(4) The statistical density/distribution plots (bottom left)	●	○	○	○
(5) The cell plot comparing all methods over time (bottom center)	○	●	○	○
(6) The time-series plots showing the evolution of the statistic over time with significance cut-off boundaries (bottom right)	○	●	○	○
(7) The aggregated coloring scheme, showing the extent to which the different methods agreed (default coloring scheme used in the maps)	●	○	○	○
The tool's design as an in-browser web-tool	●	○	○	○

Do you have any additional comments or suggestions about ClusterRadar?

selecting the area using the cursor to zoom in on the left panel (3) is very cool.

This content is neither created nor endorsed by Google.

Google Forms

ClusterRadar Feedback

This is a feedback form for ClusterRadar that should take about 5-10 minutes. If you'd like to suggest a feature or report a bug, we recommend making an issue on the GitHub repository.

If you'd like to provide some general feedback, feel free to skip to the final page of this form and fill in the "Additional comments and suggestions" box. All feedback and suggestions are really appreciated!

Background

Before using ClusterRadar, what extent were you familiar with the following concepts and techniques?

	Not familiar	Basic understanding	Working knowledge	Expert knowledge
Spatial data and analysis	○	○	○	◉
Analysis of spatial clusters	○	○	○	◉
Local spatial autocorrelation	○	○	○	◉
Hot-spot / cold-spot analysis	○	○	○	◉
Local Moran's I	○	○	○	◉
Local Geary's C	○	○	○	◉
Getis-Ord G/G*	○	○	○	◉

Do you have any additional comments about your familiarity / experience with the concepts listed in the previous question?

Sent you an email!

General Evaluation

To be filled in after using ClusterRadar. General questions about the type of analysis facilitated by ClusterRadar.

How helpful do you feel the analysis of spatial clusters would be for your work?

- ( ) Very helpful
- (•) Somewhat helpful
- ( ) Not helpful

Do you feel the analysis of spatial clusters over time provides useful additional insight over the static analysis of spatial clusters? If so, do you feel this is worth the additional complexity?

- ( ) Yes, and it is worth the additional complexity
- (•) Potentially, but it may not be worth the additional complexity
- ( ) No
- ( ) Other:

Do you feel that the simultaneous application and comparison of multiple spatial clustering methods provides useful additional insight over analysis that only employs a single method? If so, do you feel this is worth the additional complexity?

- ○ Yes, and it is worth the additional complexity
- ◉ Potentially, but it may not be worth the additional complexity
- ○ No
- ○ Other: ____________

Do you have any additional comments regarding the questions asked in this section?

____

ClusterRadar Evaluation

To be filled in after using ClusterRadar. Questions about the specific features and design decisions employed by ClusterRadar.

Do you feel that ClusterRadar achieves its primary goal of making the analysis of spatial clusters over time more accessible?

- ○ Yes
- ◉ Somewhat
- ○ No

Do you have any additional comments regarding how well ClusterRadar achieved its primary goal?

When using ClusterRadar, did you discover any interesting patterns in the data that may warrant further investigation?

How useful did you find the following features of ClusterRadar? A "detrimental" feature is one you found confusing, distracting, or otherwise detrimental to the overall experience of using the tool.

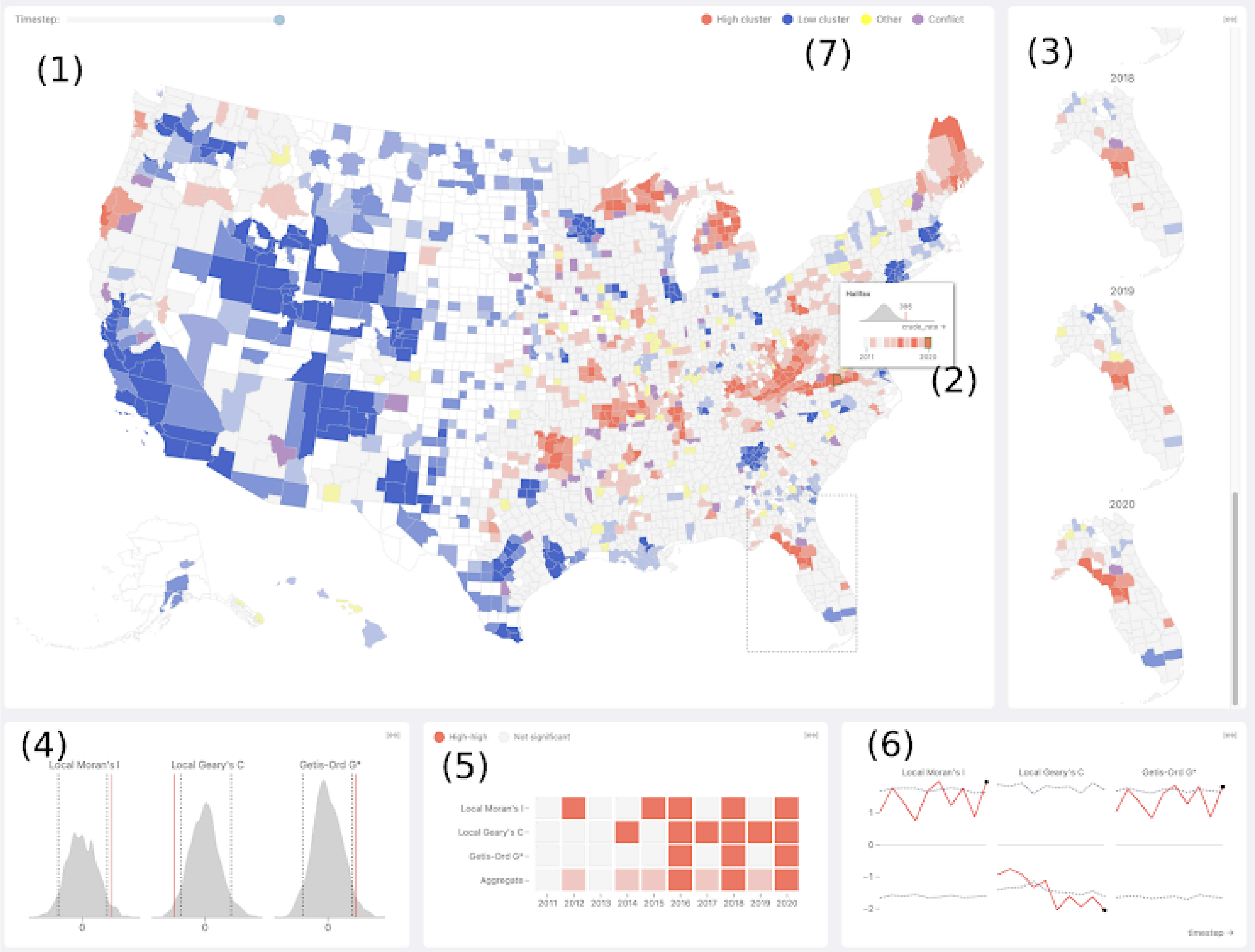

	Very useful	Somewhat useful	Not useful	Detrimental
(1) The main interactive map and time slider (the largest panel).	◉	○	○	○
(2) The graphical tooltip	◉	○	○	○
(3) The zoomed map "reel" showing the evolution of clusters over time (on the right of the page)	○	◉	○	○

(4) The statistical

(4) The statistical density/distribution plots (bottom left)	○	◉	○	○
(5) The cell plot comparing all methods over time (bottom center)	○	◉	○	○
(6) The time-series plots showing the evolution of the statistic over time with significance cut-off boundaries (bottom right)	◉	○	○	○
(7) The aggregated coloring scheme, showing the extent to which the different methods agreed (default coloring scheme used in the maps)	○	◉	○	○
The tool's design as an in-browser web-tool	◉	○	○	○

Do you have any additional comments or suggestions about ClusterRadar?

This content is neither created nor endorsed by Google.

Google Forms

ClusterRadar Feedback

This is a feedback form for ClusterRadar that should take about 5-10 minutes. If you'd like to suggest a feature or report a bug, we recommend making an issue on the GitHub repository.

If you'd like to provide some general feedback, feel free to skip to the final page of this form and fill in the "Additional comments and suggestions" box. All feedback and suggestions are really appreciated!

Background

Before using ClusterRadar, what extent were you familiar with the following concepts and techniques?

	Not familiar	Basic understanding	Working knowledge	Expert knowledge
Spatial data and analysis	○	○	◉	○
Analysis of spatial clusters	○	◉	○	○
Local spatial autocorrelation	○	○	◉	○
Hot-spot / cold-spot analysis	○	◉	○	○
Local Moran's I	○	◉	○	○
Local Geary's C	◉	○	○	○
Getis-Ord G/G*	◉	○	○	○

Do you have any additional comments about your familiarity / experience with the concepts listed in the previous question?

General Evaluation

To be filled in after using ClusterRadar. General questions about the type of analysis facilitated by ClusterRadar.

How helpful do you feel the analysis of spatial clusters would be for your work?

- ( ) Very helpful
- (x) Somewhat helpful
- ( ) Not helpful

Do you feel the analysis of spatial clusters over time provides useful additional insight over the static analysis of spatial clusters? If so, do you feel this is worth the additional complexity?

- (x) Yes, and it is worth the additional complexity
- ( ) Potentially, but it may not be worth the additional complexity
- ( ) No
- ( ) Other:

Do you feel that the simultaneous application and comparison of multiple spatial clustering methods provides useful additional insight over analysis that only employs a single method? If so, do you feel this is worth the additional complexity?

- (●) Yes, and it is worth the additional complexity
- ( ) Potentially, but it may not be worth the additional complexity
- ( ) No
- ( ) Other:

Do you have any additional comments regarding the questions asked in this section?

I really liked the ability to evaluate a group of counties and view the maps (on the right) over time. I think this will be a useful tool for visualizing trends in rates. Note it wasn't clear how to unselect the county group (to enable looking at another group).

ClusterRadar Evaluation

To be filled in after using ClusterRadar. Questions about the specific features and design decisions employed by ClusterRadar.

Do you feel that ClusterRadar achieves its primary goal of making the analysis of spatial clusters over time more accessible?

- (●) Yes
- ( ) Somewhat
- ( ) No

Do you have any additional comments regarding how well ClusterRadar achieved its primary goal?

Perhaps the article has more information but I was curious what scenarios cause the statistics to disagree.

When using ClusterRadar, did you discover any interesting patterns in the data that may warrant further investigation?

I found it interesting to see the persistent clusters over time. That seems the most useful to me.

How useful did you find the following features of ClusterRadar? A "detrimental" feature is one you found confusing, distracting, or otherwise detrimental to the overall experience of using the tool.

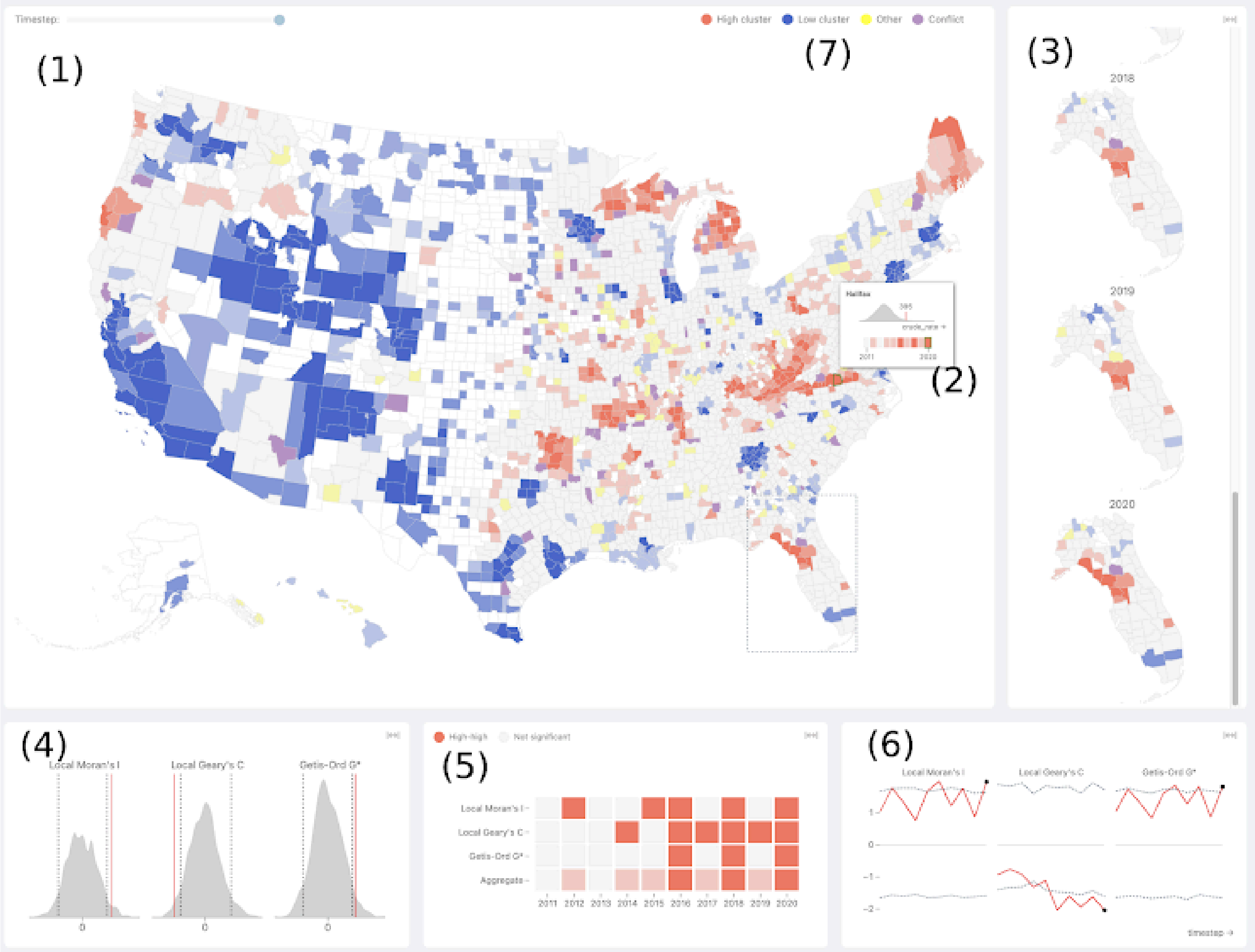

	Very useful	Somewhat useful	Not useful	Detrimental
(1) The main interactive map and time slider (the largest panel).	◉	○	○	○
(2) The graphical tooltip	○	◉	○	○
(3) The zoomed map "reel" showing the evolution of clusters over time (on the right of the page)	◉	○	○	○
(4) The statistical				

(4) The statistical density/distribution plots (bottom left)	◉	○	○	○
(5) The cell plot comparing all methods over time (bottom center)	○	◉	○	○
(6) The time-series plots showing the evolution of the statistic over time with significance cut-off boundaries (bottom right)	◉	○	○	○
(7) The aggregated coloring scheme, showing the extent to which the different methods agreed (default coloring scheme used in the maps)	◉	○	○	○
The tool's design as an in-browser web-tool	◉	○	○	○

Do you have any additional comments or suggestions about ClusterRadar?

Great job!

This content is neither created nor endorsed by Google.

Google Forms
